# Impacted and Angulated Right Hip Fracture in a Patient Reporting No History of Trauma Presenting to a Chiropractic Physician: A Case Report

**DOI:** 10.7759/cureus.51795

**Published:** 2024-01-07

**Authors:** Steven P Brown

**Affiliations:** 1 Private Practice, Brown Chiropractic and Acupuncture, Primary Care (PC), Gilbert, USA

**Keywords:** fracture, imaging, diagnosis, history, chiropractic

## Abstract

A case involving a patient with an impacted and angulated femoral neck fracture presenting to a chiropractic physician is rare. This unique case contributes an account of a challenging differential diagnosis to the literature. A 65-year-old female, reporting no history of trauma, presented with a two-week history of right lower back and right anterior hip pain radiating down the front of the right thigh in the L2-L4 dermatome. The differential diagnosis included lumbar spine disc herniation and nerve root compression or a right hip abnormality. An MRI of the lumbar spine revealed L3-L4 and L4-L5 posterior disc bulges and right foraminal narrowing. She was subsequently referred to pain management and diagnosed with lumbar radiculopathy and neural foraminal stenosis. After three lumbar spine epidural injections and a period of conservative care, the patient's symptoms were 90% improved but not fully resolved. Subsequently, right hip X-rays were ordered. The x-rays revealed an impacted and angulated right femoral neck base fracture. At this time, the patient recalled a possible traumatic incident. The patient was immediately referred to an orthopedic surgeon. After a month's delay waiting for further advanced imaging, a total right hip replacement was performed. This case underscores the importance for physicians to recognize that patients may not be aware of their own history of trauma. It also highlights the need for physicians to consider the possibility of multiple concurrent pathologies and to order imaging for all areas of pain.

## Introduction

A case involving a patient with an impacted and angulated femoral neck fracture presenting to a chiropractic physician is rare. A literature search revealed only one other case report of this nature [1]. Most patients with a femoral neck fracture typically present to the emergency department. These fractures are common, especially in patients over 65 years old in emergency settings. Femoral neck fractures can be associated with low-energy trauma in patients aged 65 and older. However, in the absence of high-energy trauma, such as from a high vertical fall, patients may not perceive their experience as "trauma" and may not report to the emergency department [2]. We present this case report to highlight that patients may not be aware of their own history of trauma, or they may have a different interpretation of "trauma." The study provides two key insights. First, it demonstrates that despite thorough history taking and subjective examinations by multiple physicians to identify prior trauma, diagnosing a traumatic injury can be challenging [3]. Second, it underscores the need for physicians to consider the possibility of multiple concurrent pathologies and that ordering imaging for all areas of pain may be clinically indicated.

## Case presentation

A 65-year-old female presented to a chiropractic physician in August 2012 with complaints of severe right lower back and anterior right hip pain, radiating from the top to the bottom of the front thigh. The onset of the pain was idiopathic and had been present for two weeks; the patient denied any injury. She speculated that she might have strained her lower back while lifting her grandchildren but could not recall a specific incident. The patient's pain varied in intensity: it was severe in certain positions and absent in others. Severe pain was elicited when she stood and attempted to raise her right foot/leg. The pain would lessen if the patient "worked through it," but at times, it was so severe that it nearly caused her to fall. The patient was limping and needed to hold onto walls for support while walking. Sitting or lying down did not trigger any pain. She was taking aspirin and 600 mg of Advil as needed every four hours but was not on any other medications and was not using ice or heat.

Upon visual analysis, the patient's gait was slow, guarded, and limping with the aid of support. Patellar reflexes were normal bilaterally, but Achilles tendon reflexes were absent bilaterally. Lower body myotome testing was normal on the left, but it was difficult to perform on the right due to pain. The Trendelenburg test was positive on the right. Bechterew's sitting test was positive on the right, while Kemp's test was negative bilaterally. The straight leg raising test was negative on both sides. Both the iliac compression test and the sacroiliac stretch test were positive bilaterally. A palpatory examination revealed myospasm and myofascial trigger points in the lumbar and right hip regions. Visual range of motion (ROM) evaluation of the lumbar spine and right hip showed decreased ROM in all planes of movement, accompanied by pain and stiffness.

The patient reported one prior episode of severe lower back pain in 2010, two years earlier. At that time, she was diagnosed with L2-L3 discitis, and her symptoms resolved within the same year. She had no further episodes of severe lower back pain until this episode in August 2012. She reported no prior instances of right hip pain. The differential diagnosis included lumbar spine disc herniation, nerve root compression, or right hip abnormality. Since there was no reported history of trauma or osteopenia/osteoporosis, lumbar spine or right hip fracture was not initially included in the differential diagnosis. Due to the pain radiating in the L2-L4 dermatome areas and the patient’s history of L2-L3 lower back disc issues, it was decided to start the evaluation by ordering an MRI of the lumbar spine to rule out lumbar disc herniation and nerve root compression. As no definitive diagnosis had been established, the chiropractic physician did not perform any treatment on the initial date of service. The patient was advised to use over-the-counter (OTC) non-steroidal anti-inflammatory drugs (NSAIDs), cold therapy, and topical anti-inflammatories at home. She was referred to a medical physician for evaluation and pain medication and was advised to visit urgent care or the ER if her symptoms worsened. It took a week for the lumbar spine MRI imaging to be performed. The MRI showed an L3-L4 posterior disc bulge and mild narrowing of both the right and left foramina. At L4-L5, there was a posterior disc bulge and mild narrowing of the right foramen. No canal stenosis or foraminal narrowing was noted at L5-S1. The patient was then referred to a pain management physician for further evaluation and treatment. Lower body myotome testing on the right remained very difficult due to pain, with the patient noting it felt more painful than weak during muscle testing. The pain management specialist diagnosed her with lumbar radiculopathy and neural foraminal stenosis.

**Figure 1:**
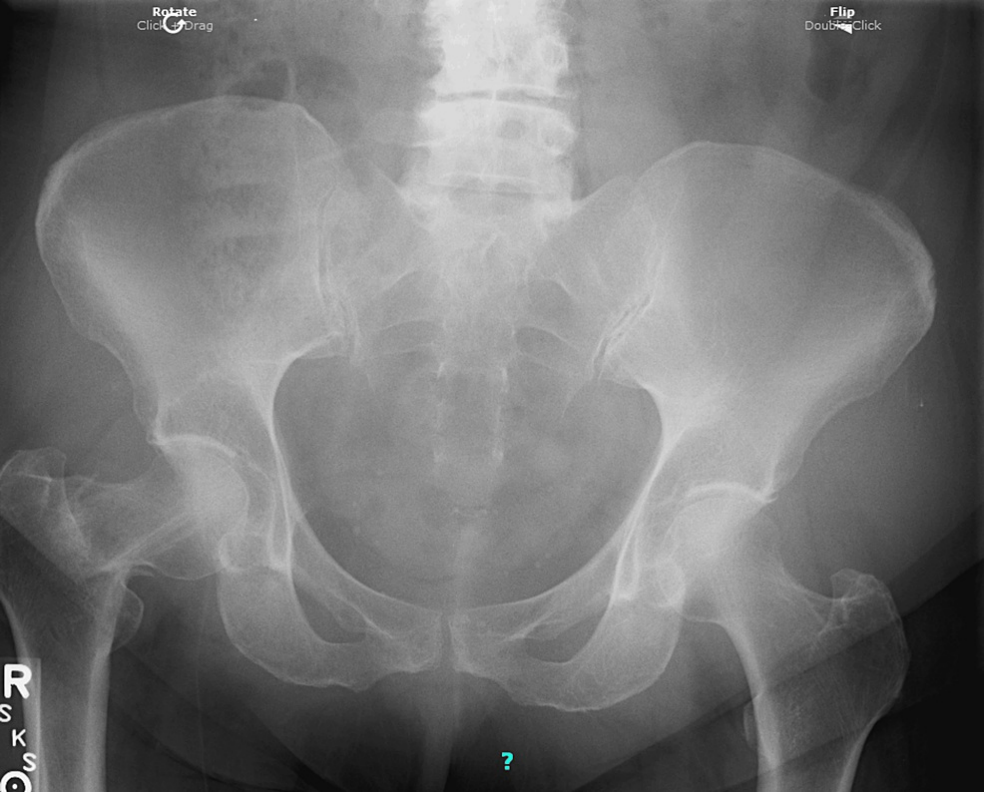
Right hip anteroposterior (AP) view.

The pain management specialist recommended transforaminal steroid injections at L3-L4. After completing three lumbar spine epidural injections, the patient reported a 90% decrease in her pain. Initially in a wheelchair before the injections, she was able to use only a cane afterward. The patient then returned to the chiropractic physician and underwent a trial of five treatments, consisting of passive physical therapy modalities, acupuncture, massage therapy, and activator spinal manipulation. No manipulation of the right hip was performed. The pain improved but was not fully resolved. The patient was able to walk without a cane after the treatments, but her pain would subsequently increase again. Due to persistent symptoms not alleviated by conservative care, the possibility of a right hip abnormality was re-evaluated at this time. Right hip X-rays were ordered, performed, and interpreted by a professional radiology facility on the same day. The X-rays revealed an impacted and angulated right femoral neck base fracture (Figure 1). The radiologist noted that the age of the fracture could not be determined from the X-ray.

Upon further questioning, the patient recalled that she unexpectedly stepped off a curb onto her right foot when the right anterior hip pain started. It is likely that this incident exacerbated her lumbar spine disc condition and caused the right hip fracture. Lifting her grandchildren may have also worsened the lumbar spine condition. The chiropractic physician immediately referred the patient to an orthopedic surgeon, who initially recommended against surgery and ordered a CT and bone scan to determine the age of the fracture [4]. Based on the patient's history, the fracture was likely over two months old at that time. It took a month for the CT and bone scan to be performed. After the imaging was completed and interpreted, immediate surgery was recommended. The patient underwent a total right hip replacement the next day. She made a good recovery with a course of post-surgical physical therapy and did not require any further chiropractic treatment or pain management. She went on to enjoy spending time with her grandchildren.

## Discussion

In this case, the patient did not recall the trauma that caused her condition. She did not perceive her unexpected step off a curb as a traumatic (low-energy fall) event that could have caused her right lower back and right hip pain. Therefore, although the chiropractic physician and the pain management specialist conducted a thorough history and inquired about prior trauma, they were unaware of this incident. The patient also consulted multiple other providers during this period. In the absence of a reported history of trauma, none of these providers considered a right hip fracture in their differential diagnosis or ordered right hip X-rays. With a known history of hip trauma in a 65-year-old female presenting with anterior right hip pain, right hip X-ray imaging to evaluate for fracture would likely have been ordered [5]. The evaluation of the patient began with ordering an MRI of the lumbar spine, as the history and examination suggested that the pain originated from the lumbar spine discs and nerve roots. However, the patient had pain in two areas: the lumbar spine and the anterior right hip. Had the physicians taken this into account and ordered right hip X-rays, the right hip fracture could have been diagnosed and treated earlier. In this case, the patient's pain was likely caused by two conditions: irritation of the right lower lumbar spine nerve roots and the right hip fracture. Including a right hip X-ray in the examination would have allowed for a timelier diagnosis of the patient's fracture. Although the chiropractic physician immediately referred the patient to an orthopedic surgeon upon diagnosing the right hip fracture via X-ray, the CT and bone scan imaging ordered by the orthopedist to determine the age of the fracture took a month to complete. During this time, the patient endured significant, unnecessary pain. The reason for the month-long delay in completing the advanced imaging for a patient with a diagnosed right hip fracture on X-ray is unclear. However, delays in obtaining advanced imaging are experiences familiar to many physicians and patients. X-rays can often be ordered, performed, and interpreted on the same day, as was the case here. However, this is not typically the situation with advanced imaging such as MRI, CT, and bone scans. A key strength of the clinical approach taken by the chiropractic physician in this case was the avoidance of manipulating the right hip. Performing manipulation on the right hip joint could have resulted in catastrophic consequences for this patient. The main weakness in the clinical approach by all physicians involved was the delay in ordering the right hip X-ray examination.

## Conclusions

This is a unique case of a patient with a right hip femoral fracture reporting no history of trauma and ambulating into a chiropractic office. This case provides two crucial takeaway insights. First, even with a thorough history and subjective examination from multiple physicians, the diagnosis of a traumatic injury can be challenging. In this case, the patient did not recall the low-energy trauma, an unexpected step off a curb, that likely caused the fracture. Patients may only associate a hip fracture with a high-energy trauma, such as a high vertical fall. Emphasizing that seemingly minor events may be traumatic may be helpful when taking a history of patients 65 and older. X-rays of all areas of pain are simple and inexpensive and can fill in gaps in clinical information that the patient may not recall. Physicians should consider that patients may have multiple concurrent pathologies, and ordering imaging of all areas of pain may be clinically indicated. The one-month delay in getting the bone scan, in this case, caused significant, unnecessary suffering for the patient and put her at risk for further injury. More immediate access to advanced imaging in the US healthcare system would lead to better patient outcomes.
